# PACE: A Probabilistic Atlas for Normal Tissue Complication Estimation in Radiation Oncology

**DOI:** 10.3389/fonc.2019.00130

**Published:** 2019-03-13

**Authors:** Giuseppe Palma, Serena Monti, Amedeo Buonanno, Roberto Pacelli, Laura Cella

**Affiliations:** ^1^Institute of Biostructures and Bioimaging, National Research Council, Naples, Italy; ^2^IRCCS SDN, Naples, Italy; ^3^Department of Engineering, University of Campania Luigi Vanvitelli, Aversa, Italy; ^4^Department of Advanced Biomedical Sciences, Federico II University School of Medicine, Naples, Italy

**Keywords:** normal tissue complication probability, voxel-based analysis, radiation-induced morbidity, radio-sensitivity, radiation therapy

## Abstract

In radiation oncology, the need for a modern Normal Tissue Complication Probability (NTCP) philosophy to include voxel-based evidence on organ radio-sensitivity (RS) has been acknowledged. Here a new formalism (Probabilistic Atlas for Complication Estimation, PACE) to predict radiation-induced morbidity (RIM) is presented. The adopted strategy basically consists in keeping the structure of a classical, phenomenological NTCP model, such as the Lyman-Kutcher-Burman (LKB), and replacing the dose distribution with a collection of RIM odds, including also significant non-dosimetric covariates, as input of the model framework. The theory was first demonstrated *in silico* on synthetic dose maps, classified according to synthetic outcomes. PACE was then applied to a clinical dataset of thoracic cancer patients classified for lung fibrosis. LKB models were trained for comparison. Overall, the obtained learning curves showed that the PACE model outperformed the LKB and predicted synthetic outcomes with an accuracy >0.8. On the real patients, PACE performance, evaluated by both discrimination and calibration, was significantly higher than LKB. This trend was confirmed by cross-validation. Furthermore, the capability to infer the spatial pattern of underlying RS map for the analyzed RIM was successfully demonstrated, thus paving the way to new perspectives of NTCP models as learning tools.

## Introduction

Early cancer detection and cancer treatment advances have contributed to improve local tumor control and overall survival. By January 1, 2024, it is estimated that in the USA the population of cancer survivors will increase to nearly 19 million individuals (1). This estimate supports research studies aimed at investigating the quality of life of these patients after the active phase of treatments, and the long-term effects of therapy.

Radiation induced damage to normal tissues is, indeed, the renowned Achille's heel of radiation treatment of cancer. The risk of normal tissue complications associated with radiation therapy may even overshadow the benefits provided in terms of tumor control, and many cancer survivors must cope with long-term effects of radiation treatments that negatively affect their quality of life. Therefore, the development of mathematical models for the estimation of Normal Tissue Complication Probability (NTCP) has long been an active field of research in order to predict the risk of radiation-induced morbidity (RIM) from the dose distribution released to critical organs (2, 3).

A major simplification traditionally applied for model-building was the introduction of the Dose-Volume Histograms (DVH) of organs at risk, designed to condense the full information of 3D dose distribution into an easy-to-handle mathematical tool, and exploited by the Lyman-Kutcher-Burman (LKB) (4) or Relative Seriality (5) models.

However, an increasing awareness has been triggered in the scientific community as to the value of including the spatial information of dose distributions within the analysis of RIM. The take-home messages of the studies that in the last 5 years have pioneered the voxel-based approach in RIM analysis fall into two categories. On the one hand, several studies highlighted an inhomogeneous radio-sensitivity of a specific organ related to the development of a given RIM (6–10); alternatively, a different application of regional analysis was proposed by (11) to allow a multi-organ study of dysphagia in head-and-neck cancer patients.

Overall, the statistical inference on spatial signature of RIM allowed to identify regions in which significant correlations exist between a global clinical outcome and the local dose release. While recognizing the urgency of including the regional inference in a planning optimization perspective, the above studies eventually resulted in identifying the mean dose to significant regions at an arbitrary α level as a RIM predictor.

Relying on such predictors might be quite unsafe, and the definition of an avoidance region based on the significant clusters of the correlation *p*-maps seems at least as simplistic as setting a single constraint on the DVH of an organ at risk. In this context, a comprehensive NTCP model able to include full spatial info on dose distribution has not yet been achieved.

The purpose of this study is to propose a new formalism to fill this gap of knowledge and to develop a Probabilistic Atlas for normal tissue Complication Estimation in radiation therapy (PACE) in order to address the need for a modern NTCP philosophy that could fit the recent voxel-based evidences on organ radiobiology. The adopted strategy basically consists in keeping the general structure of a classical NTCP model approach, such as the LKB model, and replacing the dose distribution with a collection of RIM odds as input of the model framework. The theoretical structure was first demonstrated *in silico* and then applied to a clinical dataset of thoracic cancer patients.

## Materials and Methods

### Model Design

**Figure 1:**
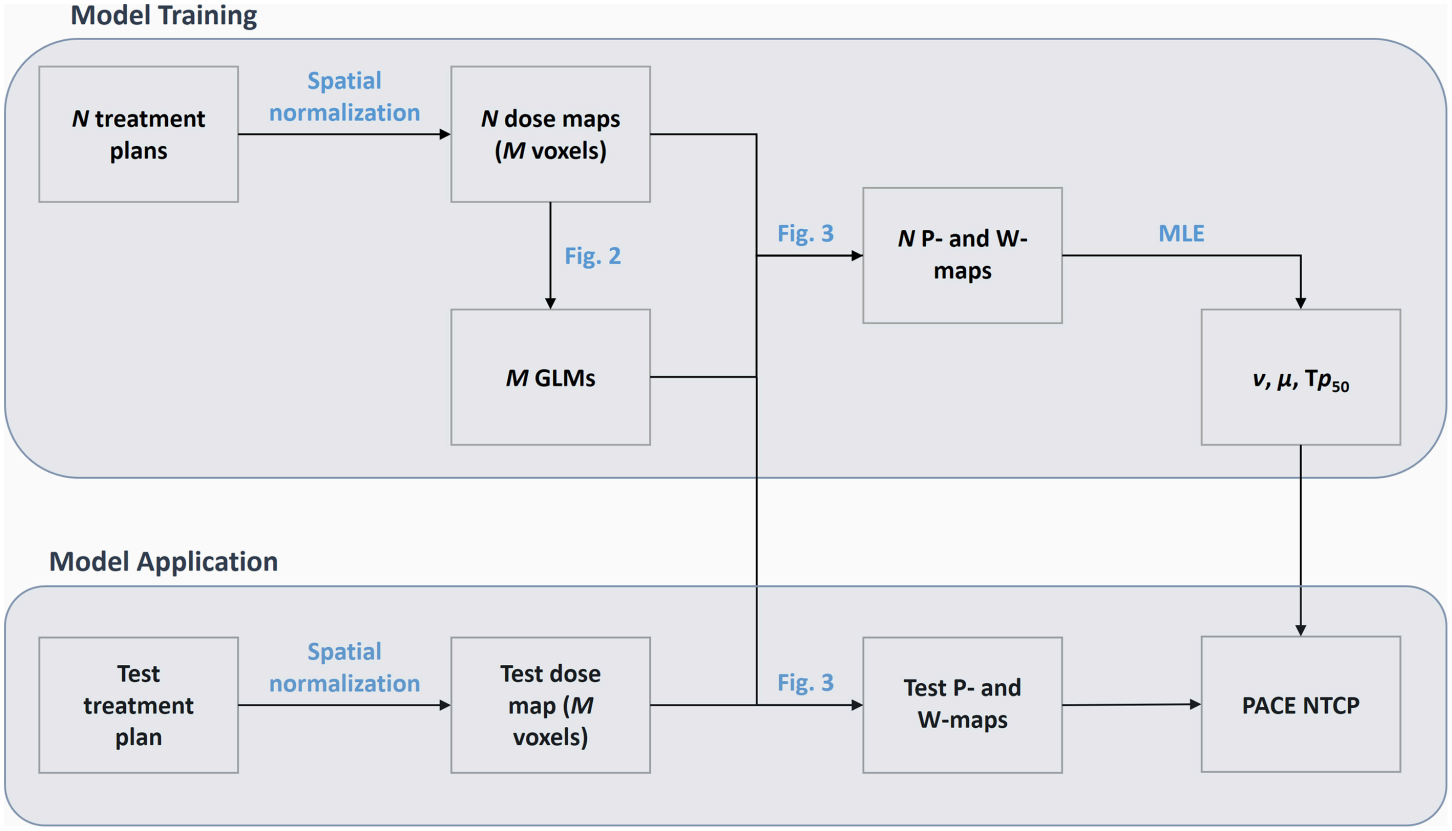
Flowchart of the PACE model. The model is first trained on a cohort of *N* patients, whose spatially-normalized dose maps are defined on a grid of *M* voxels. The training phase results in a set of *M* Generalized Linear Models (GLMs) and in the Maximum Likelihood Estimation (MLE) of the parameters ν, μ, and T*p*_50_. The application of PACE on a spatially-normalized test dose map exploit the *M* GLMs to derive a collection of global RIM predictions (*P* map) and weights (*W* map) that are finally combined according to the estimated parameters ν, μ, and T*p*_50_.

The proposed model (Figure 1) assumes the availability of a set of *N* 3D dose distributions relative to as many patients classified according to a binary global outcome associated to the considered RIM. Each dose map *D*_*i*_ (*i* = 1, …, *N*) has to be spatially normalized to a common anatomical reference in a common coordinate system. In addition, a list of possible significant non-dosimetric variables {*V*} could be available, such as global covariates {*V*_*i,k*_} (index *k* spans the non-dosimetric covariates like gender, age, medication, radiological features, etc.) or spatially normalized maps {*V*_*i,k*_(*x*_*j*_)} (CT, MRI, PET, etc.) defined on each voxel *x*_*j*_.

**Figure 2:**
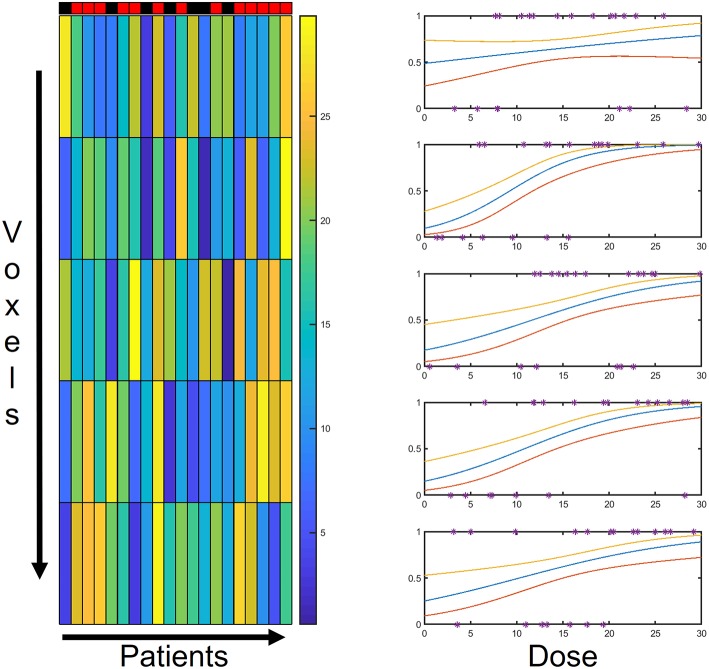
Schematic representation of model backbone: for each voxel, the *N* patients' outcomes are tied to the local dose through a logistic regression. The PACE structure relies on as many logistic regression models as the number of voxels.

For each voxel, a logistic regression of the *N* outcomes is performed based on the *N* values of local dose {*D*_*i*_(*x*_*j*_)|*i* = 1, …, *N*} (Figure 2) and of each covariate {*V*_*i,k*_(*x*_*j*_)|*i* = 1, …, *N*}.

**Figure 3:**
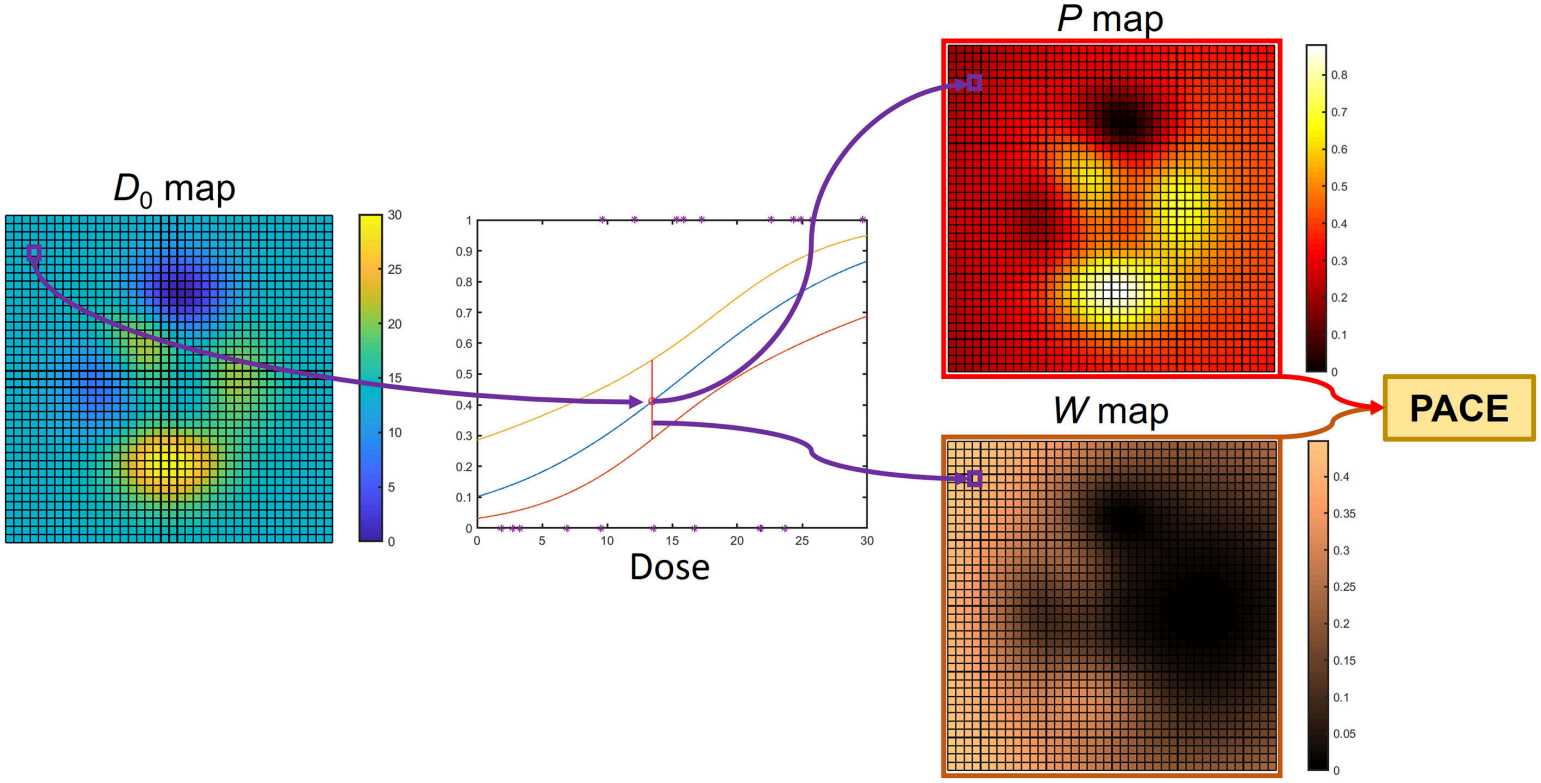
Computation of PACE prediction on a test patient with dose map *D*_0_: for each voxel, the regression model collected in the model backbone (Figure 2) is exploited to guess a RIM risk and the associated confidence interval. Thus, a collection of global RIM predictions (*P* map) and weights (*W* map) are populated voxelwise. These are properly merged to produce the actual PACE probability.

The computation of PACE prediction on a test patient with a dose map *D*_0_ is then obtained as follows (Figure 3). For each voxel, the corresponding regression value *P*(*x*_*j*_) and the associated 95% confidence interval CI(*x*_*j*_) are evaluated based on the local *D*_0_(*x*_*j*_) and *V*_0, k_(*x*_*j*_). Thus, a collection of global RIM predictions *P*(*x*_*j*_) is populated voxelwise, along with the associated reliability scores given by *W*(*x*_*j*_) = 1/CI(*x*_*j*_), where the odds ratio for the dose is < 1 (i.e., dose appears to be protective), *W*(*x*_*j*_) = 0. The overall PACE prediction is then summed up in a LKB fashion as

(1) $$\text{PACE}=\frac{1+\mathrm{erf}\frac{t}{\sqrt{2}}}{2},$$

where

(2) $$t=\frac{\text{gEU}p-\text{T}p_{50}}{\mu \cdot \text{T}p_{50}}$$

and gEU*p* (generalized equivalent uniform probability) is defined as

(3) $$\text{gEU}p={\left[\frac{\sum_{j}p^{\frac{1}{v}}\left(x_{j}\right)W\left(x_{j}\right)}{\sum_{j}W\left(x_{j}\right)}\right]}^{\nu }.$$

The model contains three free parameters–namely ν (equivalent to the volume effect parameter *n*), μ (controlling the slope of the response sigmoid curve) and the tolerance probability T*p*_50_–whose values can be customarily derived for a particular RIM by maximizing the likelihood function of the PACE model (12) [the related confidence interval are estimated according to Wilks' theorem for nested models (13)].

### *In silico* Validation

PACE model has been first demonstrated on two classes of sets (of increasing cardinality *N*) of 2D synthetic dose maps defined on a square region of interest.

**Figure 4:**
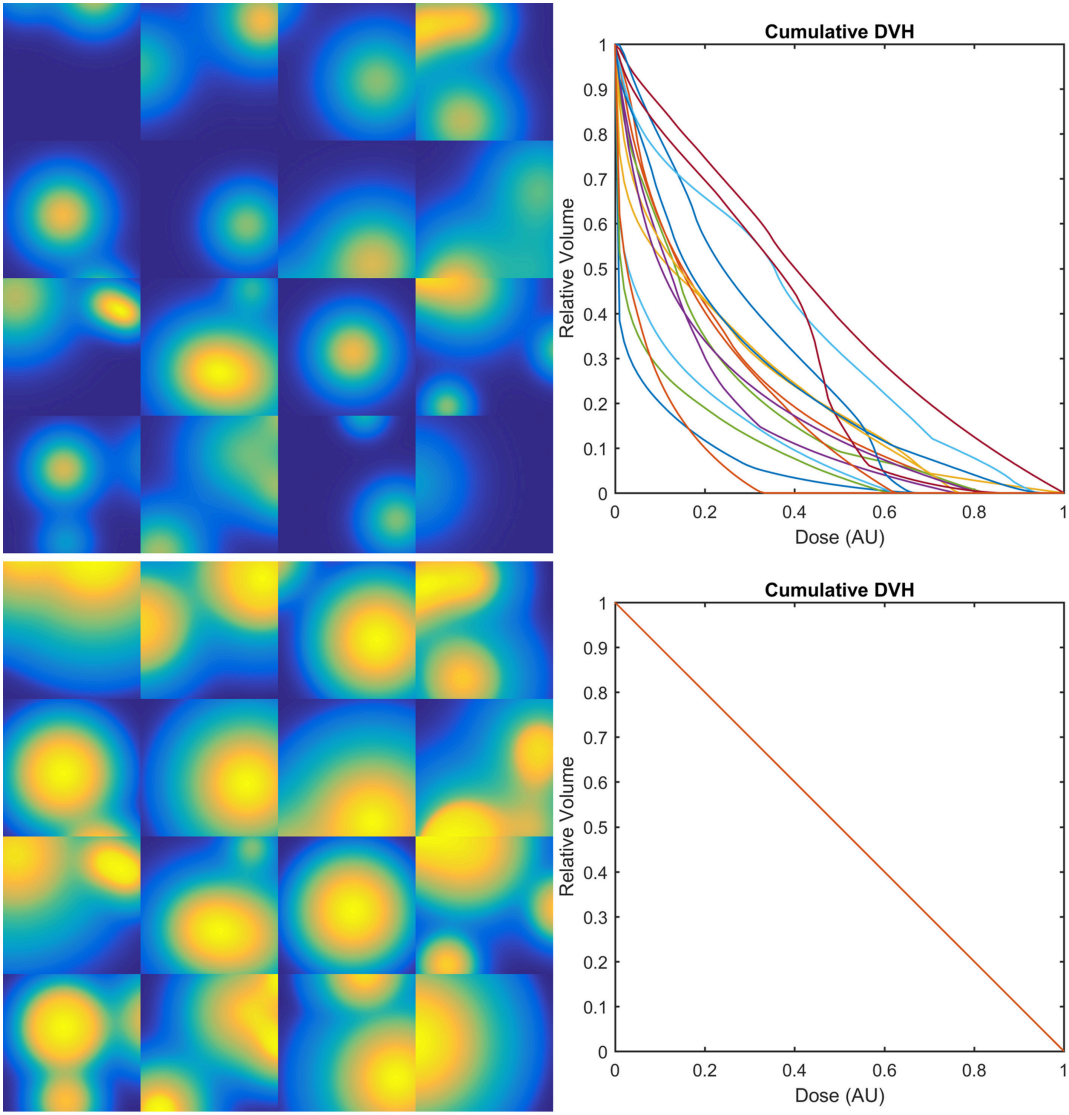
Examples of 2D synthetic dose maps (on the left) and corresponding Dose-Volume Histograms (DVHs–on the right): upper row refers to dose distributions generated as the sum of a random number (from 1 to 4) of Gaussian peaks whose standard deviation ranges from 10 to 30% of the size of the region of interest and whose height ranges from 0.5 to 1.5 in arbitrary units (AU); lower row refers to the same dose maps transformed in order to provide the same DVH for all the dose distributions.

The dose distributions *D*_*i*_(*x*_*j*_) of the first class of sets were generated as the sum of a random number (from 1 to 4) of Gaussian peaks whose standard deviation ranges from 10 to 30% of the size of the region of interest and whose height ranges from 0.5 to 1.5 in arbitrary units (Figure 4, upper row).

The second class was obtained from the first one by transforming the original dose maps as *E*_*i*_(*x*_*j*_) = *g*_*i*_[*D*_*i*_(*x*_*j*_)], where each *g*_*i*_ is defined in order to provide the same constant differential DVH on a compact support for all the *E*_*i*_ (Figure 4, lower row).

**Figure 5:**
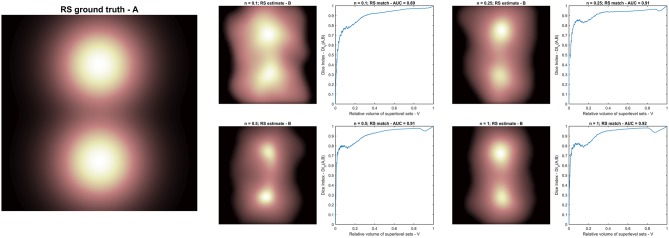
Radio-sensitivity (RS) map inference: **(A)** RS ground truth; **(B)** RS estimation in arbitrary units from different synthetic radiobiologies obtained for increasing volume effect parameter *n*. Each RS estimate is supported by the plot of the match metric (DI_*V*_).

To classify the dose maps according to a realistic synthetic outcome, a new summary statistic, derived from the generalized Equivalent Uniform Dose (14), was designed for a given dose distribution *D*(*x*_*j*_) as

(4) $${\text{g}}^{2}\text{EUD}={\left[\frac{\sum_{j}D^{\frac{1}{n}}\left(x_{j}\right)\text{RS}\left(x_{j}\right)}{\sum_{j}\text{RS}\left(x_{j}\right)}\right]}^{n}$$

in order to account for possible inhomogeneities in organ radio-sensitivity (RS), besides the usual volume effect parametrized by the organ dose-volume effect parameter *n* (2). For each class of dose maps, two RS maps were considered: a homogeneous one and one given by the sum of two shifted Gaussian distributions (S2G–Figure 5A). The g^2^EUDs were then compared to a threshold value *D*_th_, thus deriving a synthetic RIM outcome as O_*i*_ = [g^2^EUD_*i*_>*D*_th_]. Of note, the second class of dose sets was not evaluated according to a homogeneous RS, since the maps would have been indistinguishable.

Each set of classified dose distributions was then split in a training and a validation set, and the learning curves (15) of PACE and LKB (2) models were finally compared.

### Application to Clinical Data

The PACE model was applied to a cohort of *N* = 98 thoracic cancer patients classified for lung fibrosis of any grade according to RTOG late pulmonary toxicity scoring system (18 events) (16). The age was the only non-dosimetric variable significantly correlated (*p* = 0.019) with the considered RIM (17). All participants gave their written informed consent and patient data were analyzed anonymously. This retrospective study was approved by the local Ethics Committee (Comitato Etico per le Attività Biomediche, Università “Federico II,” Napoli, n. 222–10). All experimental protocols and procedures were performed in accordance with the guidelines of the Università “Federico II,” Napoli.

To satisfy PACE assumptions, each dose map was normalized to a common anatomical reference via a log-diffeomorphic demons registration tool, as described in Palma et al. (8) and Monti et al. (17).

The full PACE model including dose maps and age, the purely dosimetric PACE (18) and the LKB models were trained for comparison.

Model performances were evaluated by the Area Under the Receiver Operating Characteristic (ROC) Curves (AUCs) and via the calibration plots. ROC-AUCs were compared by a Z-test according to the standard error estimates provided by (19). A Leave-One-Out (LOO) cross validation of the models was performed. The accuracy (i.e., [True Positive + True Negative]/[ Positive + Negative]), the balanced accuracy (i.e., [True Positive/Positive + True Negative/Negative]/2) and the *F*_1_ score (i.e., 2^*^True Positive/[2^*^True Positive + False Positive + False Negative]) of predictions were computed.

### Radio-Sensitivity Mapping

A spinoff application of the proposed approach was evaluated. Such application can lead beyond the strict sense of NTCP and potentially discloses new insights on RS maps. In particular, once a PACE model has been trained on a given dataset, the level sets of the RS map can be inferred by computing PACE predictions on new purposely generated probe-set of synthetic dose distributions. The latter are designed with a single, narrow hot spot, whose position shifts along the set to span the entire region of interest. The PACE probability obtained for a given dose distribution of the probe-set can be then related to the position of the associated hot spot, thus getting a voxelwise measure of the underlying RS map.

The RS map inference was therefore evaluated on four PACE models; each model was trained on a dataset of 1,000 dose maps classified according to Equation 4 with a different value of the volume effect parameter *n* and using the same S2G as RS map (Figure 5).

In order to quantify the match between the RS ground truth (*A*) and each *a posteriori* estimate (*B*), the following metrics is defined as the Dice Index (DI) (20) between the superlevel sets of *A* (*S*_*V*_[*A*]) and *B* (*S*_*V*_[*B*]) with same size *V* (17):

(5) $${\text{DI}}_{V}\left(A,B\right)=\text{DI}\left(S_{V}\left[A\right],S_{V}\left[B\right]\right)$$

The NTCP models as well as the evaluation steps were implemented in house in Matlab (MATLAB® Release 2016b, The MathWorks, Inc., Natick, MA, USA).

## Results

### *In silico* Performances

**Figure 6:**
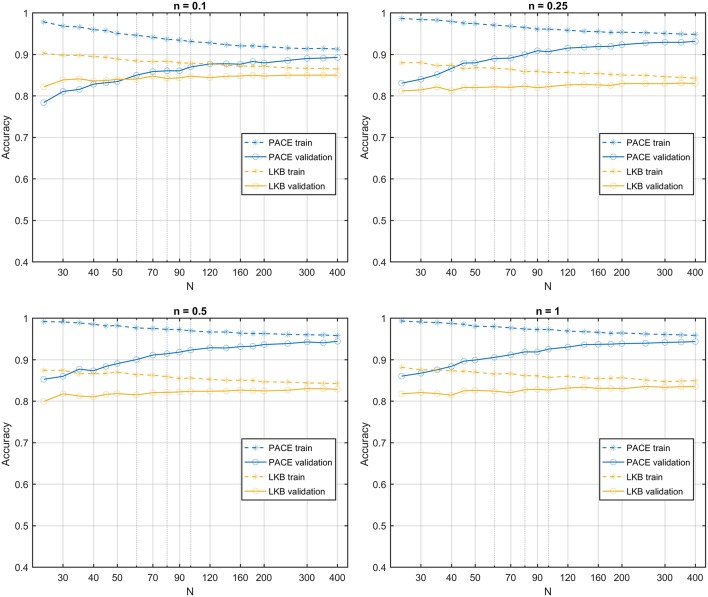
Learning curves for PACE and LKB models on sum of two shifted Gaussian radio-sensitivity distribution for increasing volume effect parameter *n*.

**Figure 7:**
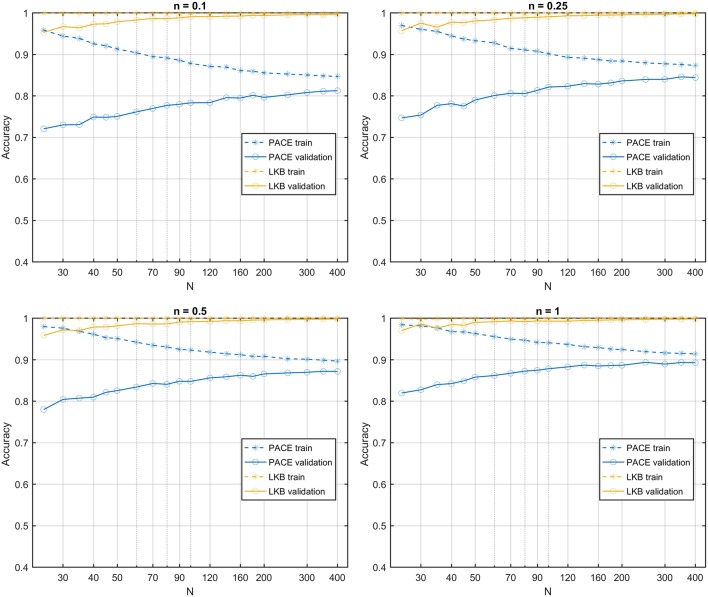
Learning curves for PACE and LKB models on homogeneous radio-sensitivity distribution for increasing volume effect parameter *n*.

The learning curves obtained on the first class of datasets classified according to a S2G RS map show that the PACE model outperforms the LKB model (Figure 6), with a slightly increasing model bias at lower *n* values. When the same class of datasets is ranked according to a homogeneous RS map, by construction, the LKB model turns out to be, the perfect classifier (Equation 4 coincides with standard gEUD definition). Nonetheless, PACE proves still able to provide an accuracy around the 0.8 level (Figure 7).

**Figure 8:**
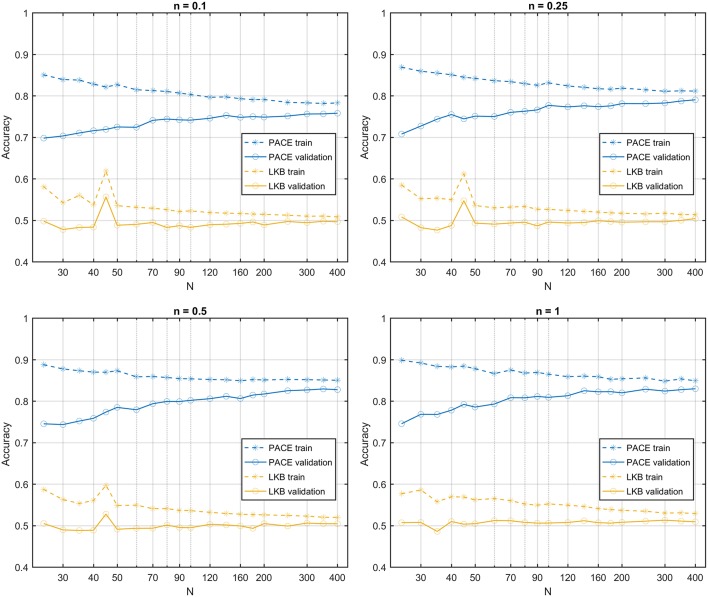
Learning curves for PACE and LKB models on sum of two shifted Gaussian radio-sensitivity distribution for increasing volume effect parameter *n*; dose distributions have identical Dose-Volume Histogram.

In the second class of datasets, all dose maps are summarized by the same DVH. Therefore, the LKB model turns out to be a random classifier (expected accuracy = 0.5), while the PACE model still predicts the outcome with an accuracy >0.8 (Figure 8).

### Clinical Data Demonstration

The training process of the considered models on the real patients' data was summarized in Table 1.

**Table 1:** Training and validation of PACE, PACE_NoAge_, and LKB models on clinical data: Parameters and performance scores.

	**PACE**		**PACE**_****NoAge****_		**LKB**	
**Parameters (95% CI)**	ν	0.05 ([0.01–0.10])	ν	0.12 ([0.01–0.25])	*n*	0.06 ([0.01–1])
	μ	0.39 ([0.29–0.55])	μ	0.34 ([0.20–0.54])	*m*	0.25 ([0.13–0.98])
	T*p*_50_	0.55 ([0.42–0.76])	T*p*_50_	0.38 ([0.25–0.75])	TD_50_	36 ([18–300]) Gy
Discrimination value	0.37		0.10		0.20	
**Performance**						
AUC (95% CI)	0.85 ([0.76–0.91])		0.79 ([0.70–0.87])		0.66 ([0.56–0.76])	
Calibration slope ± SE	1.06 ± 0.13		0.92 ± 0.14		0.76 ± 0.39	
Calibration intercept ± SE	−0.010 ± 0.037		−0.012 ± 0.039		0.012 ± 0.078	
Calibration *R*^2^	0.93		0.90		0.50	
LOO Accuracy	0.74		0.82		0.70	
LOO Balanced accuracy	0.67		0.65		0.62	
LOO *F*_1_	0.44		0.44		0.38	
LOO AUC (95% CI)	0.75 ([0.65–0.83])		0.67 ([0.57–0.76])		0.59 ([0.48–0.68])	
LOO Calibration slope ± SE	0.56 ± 0.11		0.55 ± 0.14		0.29 ± 0.24	
LOO Calibration intercept ± SE	0.063 ± 0.036		−0.061 ± 0.045		0.103 ± 0.047	
LOO Calibration *R*^2^	0.83		0.77		0.43	

*CI, confidence interval; AUC, Area Under the ROC Curve; SE, Standard Error; LOO, Leave One Out*.

The models consistently highlighted a similar volume effect for the organ response to irradiation (see ν and *n* parameters); however, the confidence intervals of the three free parameters estimated for PACE models were systematically smaller than the LKB ones.

**Figure 9:**
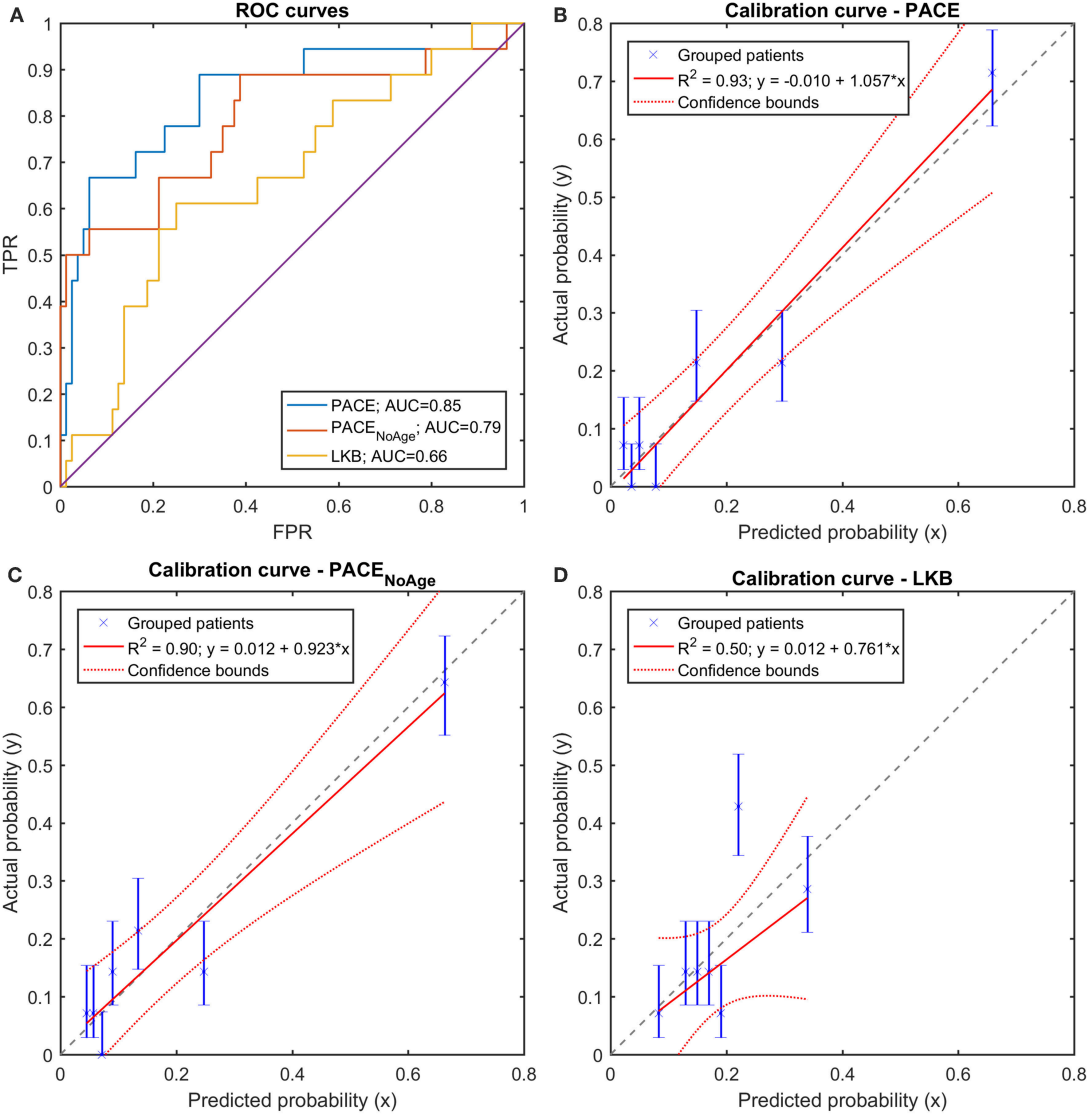
**(A)** Receiver Operating Characteristic (ROC) curves and **(B–D)** Calibration curves for the three trained models (full PACE, purely dosimetric PACE, and Lyman-Kutcher-Burman–LKB) on the clinical dataset.

On the training set, both PACE models outperformed the LKB model in terms of discrimination (Figure 9A): at a pairwise comparison with the LKB ROC curve, the full PACE model showed an AUC significantly higher (*p* = 0.018), while a trend was found for the purely dosimetric PACE model (*p* = 0.054). The calibration curves of both PACE models show higher *R*^2^ compared to LKB, with calibration-in-the-large *a* and calibration slope *b* close to the ideal (i.e., *a* = 0 and *b* = 1), in contrast to LKB (Figures 9B–D).

**Figure 10:**
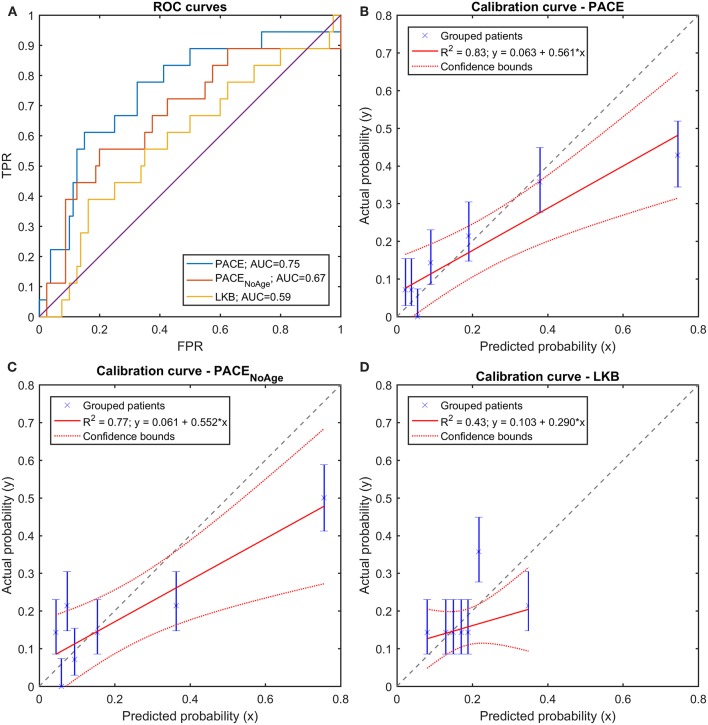
**(A)** Receiver Operating Characteristic (ROC) curves and **(B–D)** Calibration curves for the three trained models (full PACE, purely dosimetric PACE, and Lyman-Kutcher-Burman–LKB) on the Leave-One-Out cross-validation on the clinical dataset.

A LOO cross validation confirmed the better performances of PACE models compared to LKB model (Figure 10A), as shown by the accuracy and balanced accuracy of outcome predictions. Furthermore, the above described patterns of discrimination and calibration survived at validation (Figures 10B–D).

### Radio-Sensitivity Inference

The RS maps probed with the PACE models trained on the synthetic datasets suggest a trend in the spatial pattern for increasing *n*. Nonetheless, the AUC of DI_*V*_ between inferred and ground truth RS maps (overall close to 0.9) reveals that the shape of the level sets is only mildly dependent on *n* (Figure 5).

## Discussion

We devised a mathematical solution for the statistical modeling of NTCP that allows to deal with inhomogeneous organ susceptibility to radiation exposure. This was solicited by the recent evidences from voxel-based analyses and by two-decade studies about non-local dose effects on tissue damages (21).

In the last ten years several attempts have been made to include heterogeneous dose-response relationships into NTCP models.

Some studies highlighted a spatially dependent point-to-point correlation between dose and induced damage, which, in the context of lung toxicity, could be indexed by the variation of CT density (accounting for fibrosis) (22, 23) or of SPECT activity (measuring the perfusion) (24). Despite the value of such findings, the above studies were not suggesting an actual NTCP model, since no global outcome was related to the predicted local radiological variations.

Differently, Dean and coworkers (25, 26) included spatial dose metrics (3D moment invariants) of the oral mucosa within their multivariable analysis of severe acute mucositis, thus accounting for possible regional variations in RS through some summary parameters. In a following study on dysphagia resulting from head-and-neck radiation therapy (27), they found that such approach did not add any significant predictive power to the DVH analysis, possibly due to the high correlation between such spatial metrics and the ordinary DVH metrics. Limited to the prediction of rectal bleeding following prostate cancer radiation therapy, an ensemble of neural networks was trained in (28) to find the relevant features in a 2D dose-surface map of the rectum. Similarly, in (29) it is shown that the predictability of gastro-intestinal toxicity increases using spatial metrics compared to DVH metrics. Furthermore, some studies on dose map population comparison highlighted a somehow strong correlation between the RIM outcome and the mean dose to a sublevel set of the significance *p*-map (7, 8, 11). However, such statistics could be barely robust predictors of toxicity since they are intrinsically unable to account for differently significant spatial dose signature of RIM.

The proposed model falls within the large framework of the phenomenological models, which, in opposition to mechanistic models (30), aim at a substantial consistency with the available data without necessarily relying on a fully established radiobiological background (31).

The functional form of the PACE model was designed to take into account some key points. First, a huge body of literature on NTCP suggests that the radiation toxicity is a function more or less related to the extent of the dose release, depending on structure and physiology of the organ and on the considered endpoint. Second, same doses to different subregions could in principle result in different odds of global damage. Finally, the relationship between dose and outcome can be more or less strong within the considered anatomical district.

The first point suggested to adopt a theoretical framework including a parameter for the description of the dose-volume effect, such as the largely exploited DVH-based LKB model (Equation 1). The second point led to replace the dose distribution with a collection of odds specifically estimated for the spatial position of local dose release (Equation 2). Finally, the third point was addressed by weighting the odds according to their uncertainty (Equation 3).

The PACE framework, by construction, allows to naturally model a multivariable phenomenon such a RIM, by including possible non-dosimetric covariates (potentially point functions) within the assessment of NTCP.

The model was first demonstrated *in silico*. To this purpose, we devised and implemented several synthetic plausible radiobiologies, in order to simulate diverse RS maps and several dose-volume effects. PACE scheme was able to learn the responses assigned to the dose distributions with high accuracy and robustness, consistently outperforming the LKB approach in a wide range of synthetic physiopathologies. Not surprisingly, the reported learning curves revealed that, compared to LKB, the PACE model requires a higher number of dose distributions to reduce the deviation between training and validation accuracies, as a result of the wider spectrum of information it has to take into account. Nevertheless, the overall lower model bias exhibited by PACE allows to obtain more accurate predictions in the validation set even at moderately low cardinality (*N* < 50) of the training set.

This is reflected in the application to a real clinical dataset of about 100 patients with a relatively low number of RIM events (18%). The estimates of the volume-effect parameters (ν for PACE or *n* for LKB) highlighted the relevance of the value of the highest doses in the RIM prediction, which is consistent with some previous findings on lung toxicity (16, 32). Interestingly, the same dataset permits to obtain much sharper estimates of the PACE model parameters than those provided by the LKB.

PACE performance, evaluated by both discrimination and calibration scores, was significantly higher than the LKB benchmark. This trend was confirmed by the cross-validation, which ruled out severe overfitting issues, despite the complexity of the PACE structure.

It is worth mentioning that the better accuracy achieved by the full PACE model is not to be ascribed just to the inclusion of a significant clinical covariate (i.e., the age). Indeed, while the PACE superiority bears witness to the opportunity to incorporate non-dosimetric variables, the scores obtained by the simpler PACE_NoAge_ still prove a sensible improvement over a conventional NTCP scheme.

Remarkably, a further application of this new NTCP philosophy concerns the capability to infer the spatial pattern of underlying RS map for the analyzed RIM. The fulfillment of such task was successfully demonstrated for several synthetic radiobiologies, showing that the RS mapping tool applies pretty well over a wide spectrum of dose-volume effect behavior driven by *n*. This enables a change in perspective for the usage of NTCP models, which turns out to be a valuable learning tool for knowledge building if properly queried. A key point in this context, strictly related to the generalizability of the PACE model, are the characteristics of the learning cohort of patients, which—similarly to what happens in the training of standard DVH-based NTCP models—is warranted to show highly inhomogeneous and mutually uncorrelated dose distributions (11).

At the same time, the very nature of this new NTCP scheme, which takes into account the full 3D distribution of dose, makes it particularly suitable for studying toxicity outcomes related to modern radiation therapy techniques, such as stereotactic body radiation therapy and particle therapy, characterized by greatly heterogeneous dose distributions.

We are aware that the increased complexity of a PACE model, compared to the corresponding LKB function, may hinder the diffusion of this scheme in the clinical practice. Nonetheless, an increasing consciousness in the radiation oncology community of the inhomogeneous RS of several organs justifies and actually calls for an adequate and modern NTCP approach. On the other hand, PACE scheme can be considered in all respects within the large family of machine learning algorithms, which find more and more room within the recent releases of treatment planning systems.

In conclusion, we designed and demonstrated, both *in silico* and in clinical datasets, an original, phenomenological NTCP model, which we nicknamed PACE, to blend the need for RIM prediction with the awareness of inhomogeneous dose susceptibility. It is conceived to work for different RIM outcomes from arbitrary district irradiation, with minimal research cognitive bias. We believe this could open new perspectives for clinical radiobiology.

## Data Availability

The datasets analyzed for this study can be found in http://www.ibb.cnr.it/?command=viewcms&and2=32&id=226.

## Author Contributions

GP and LC conceived and designed the study. RP participated in patient recruitment and collected the clinical data. GP wrote the code. GP, SM, and LC processed the data. GP and AB analyzed the results. GP and LC wrote the manuscript. All authors reviewed the final manuscript.

### Conflict of Interest Statement

The reviewer MD declared a past co-authorship with one of the authors LC to the handling Editor. The remaining authors declare that the research was conducted in the absence of any commercial or financial relationships that could be construed as a potential conflict of interest.
